# Calcium Hydroxyapatite and Polymicronutrient Solution on Hand Rejuvenation: A Split‐Hand, Randomized, Double‐Blind Clinical and In Vitro Study

**DOI:** 10.1111/jocd.70716

**Published:** 2026-02-11

**Authors:** Gladstone Eustáquio de Lima Faria, Cibele Hasmann, Renata M. M. Viana, Ana Carolina Henriques Ribeiro Machado, Beatriz Domenici de Oliveira, Rebecca Ignácio Subirá Medina, Agnaldo Castro Filho, Luciana Zattar, Gláucia M. Machado‐Santelli, Ricardo Frota Boggio

**Affiliations:** ^1^ Instituto Boggio São Paulo São Paulo Brazil; ^2^ Private Practice São Paulo São Paulo Brazil; ^3^ Ilikia São Paulo São Paulo Brazil; ^4^ Universidade de São Paulo São Paulo São Paulo Brazil

## Abstract

**Background:**

Calcium hydroxyapatite (CaHA) is a well‐established biostimulatory filler approved for hand rejuvenation. Recent approaches have explored dilution with polymicronutrient (PMN) solutions to enhance cellular metabolism and extracellular matrix (ECM) regeneration.

**Aims:**

To evaluate the biological and clinical effects of CaHA diluted in a PMN solution (CaHA + PMN) compared with the conventional CaHA diluted in saline solution (CaHA + SS) for hand rejuvenation.

**Methods:**

An in vitro study was conducted to assess fibroblast proliferation and gene expression of ECM components (COL1A1, COL3A1, and ELN) after exposure to CaHA diluted with PMN or SS. A prospective, split‐hand, double‐blind clinical trial (*n* = 22) compared both formulations regarding Hand Grading Scale (HGS), Global Aesthetic Improvement Scale (GAIS), skin hydration (corneometry), and dermal thickness (ultrasound imaging) at baseline, 15 and 90 days after treatment.

**Results:**

In vitro, CaHA + PMN induced greater fibroblast proliferation and upregulated COL1A1 and ELN gene expression compared to CaHA + SS. Clinically, both treatments led to significant improvement from baseline in HGS (*p* < 0.001), skin hydration and dermal/hypodermal thickness, with no statistically significant differences between‐group. Investigator‐assessed GAIS and patient‐reported satisfaction on a 5‐point Likert scale also showed improvement in both groups.

**Conclusion:**

Both treatments demonstrated comparable clinical outcomes, suggesting that the strong biostimulating effect of CaHA may have overshadowed potential additive effects of PMN. Nonetheless, in vitro findings confirmed enhanced biological activity with CaHA + PMN, supporting its investigation as a complementary strategy in future studies.

## 1 Introduction

Hand rejuvenation has become an increasingly popular aesthetic procedure designed to reverse the visible signs of aging on the dorsum of the hands. Aging‐related natural loss of subcutaneous fat and collagen leads to volume loss, decreased elasticity, and dermal thinning, which contribute to the prominence of veins and tendons [1]. Non‐invasive and minimally invasive treatments are effectively used to restore volume, smooth out wrinkles, and enhance the overall appearance of the hands. These treatments include dermal fillers, mesotherapy serums, biostimulators, lasers, and regenerative solutions [2, 3, 4]. Each of these modalities acts through different mechanisms to improve hand aesthetics, addressing various aspects of aging and contributing to a more youthful appearance.

Among these treatments, calcium hydroxyapatite (CaHA) is a well‐established and widely used agent in aesthetic medicine, recognized for its safety and efficacy [4]. Since 2015, CaHA has been approved by the U.S. Food and Drug Administration (FDA) also for hand rejuvenation, underscoring its reliability in this indication. The mechanism of action of CaHA is twofold: (1) it acts as a dermal filler providing immediate volume through its carboxymethylcellulose‐based gel, and (2) it serves as a long‐term biostimulation. Diluted CaHA promotes fibroblast proliferation, and stimulates elastin and collagen synthesis, leading to skin texture and elasticity improvement over time [5].

To optimize the biostimulatory effects of CaHA, it has increasingly been combined with polymicronutrient (PMN) solutions. The rationale behind this combination lies in the concept that aging fibroblasts may benefit from a direct supply of essential biosynthetic substrates, which could restore their synthetic capacity and enhance dermal regeneration, particularly in aged or metabolically impaired skin. Emerging evidence suggests that priming the skin with a PMN solution may potentiate the overall response to CaHA treatments by providing essential cofactors such as amino acids, vitamins, and coenzymes for optimal fibroblast function and extracellular matrix (ECM) protein synthesis [6].

In vitro studies have demonstrated that polycomponent mesotherapy formulations significantly enhanced fibroblast proliferation and stimulated both intracellular and extracellular collagen synthesis in normal and aged fibroblasts, compared to untreated controls [7]. This approach has more recently been described as “skin priming,” a strategy aimed to preparing the tissue microenvironment by enriching it with bioactive nutrients to enhance the regenerative response to biostimulatory agents like CaHA [6].

This study was designed to comprehensively evaluate the biological and clinical effects of combining CaHA with a PMN solution. To this end, two complementary arms were conducted: (1) a randomized, double‐blind clinical trial assessing efficacy, safety, and patient satisfaction in dorsal hand rejuvenation; and (2) in vitro assays investigating fibroblast proliferation and gene expression of ECM components. Both datasets were integrated to provide a robust mechanistic and clinical understanding of the treatment strategy.

## 2 Materials and Methods

We tested a CaHA dermal filler (STIIM by Ilikia, CG Bio Co. Ltd., South Korea), consisting of 30% calcium hydroxyapatite (CaHA) microspheres suspended in 70% carboxymethylcellulose gel. The PMN solution used was Pluryal Refresh (MD Skin, Luxembourg), a product containing a blend of PMNs (listed in Table S1), including non‐cross‐linked hyaluronic acid (HA) at a concentration of 10 mg/mL.

### 2.1 In Vitro Analysis

#### 2.1.1 Cell Culture and Experimental Design

Primary human dermal fibroblasts (HDFs) were cultured in high‐glucose Dulbecco's Modified Eagle Medium (DMEM; Gibco), supplemented with 10% fetal bovine serum (FBS; Gibco) and 1% penicillin–streptomycin. Cells were maintained at 37°C in a humidified atmosphere containing 5% CO_2_ and used between passages 4 and 6 for all experiments.

For the assays, fibroblasts were seeded at a density of 1 × 10^4^ cells per well in 96‐well plates (for proliferation analysis) or 6‐well plates (for gene expression analysis) and allowed to adhere before treatment.

Twelve experimental groups were established to evaluate the biological effects of CaHA diluted in either saline or PMN solution, as well as the isolated components and untreated controls. A summary of the experimental groups, including treatment composition and dilution ratios, is presented in Table S2. While the 1:1 dilution reflects the clinical protocol, additional ratios (1:2 to 1:4) were included to explore dose‐dependent cellular responses.

Test formulations were prepared by manual mixing immediately before application. All experimental conditions were tested in triplicate and repeated in independent runs.

#### 2.1.2 Cell Proliferation Assay (MTT)

After 72 h of incubation with the test formulations, cell viability and proliferation were assessed using the MTT assay (3‐[4,5‐dimethylthiazol‐2‐yl]‐2,5‐diphenyltetrazolium bromide; Sigma‐Aldrich). This colorimetric assay is based on the reduction of MTT by mitochondrial dehydrogenases in metabolically active cells, resulting in the formation of purple formazan crystals.

A volume of 10 μL of MTT solution (5 mg/mL in PBS) was added to each well, followed by incubation at 37°C for 4 h. After this period, the medium was removed, and the formazan crystals were solubilized with 100 μL of dimethyl sulfoxide (DMSO). Absorbance was measured at 570 nm.

#### 2.1.3 Gene Expression Analysis

Gene expression analysis was performed to evaluate the modulation of ECM components in fibroblasts after exposure to the test formulations. Cells were treated for 72 h and then processed for RNA extraction using the RNeasy Mini Kit (Qiagen) according to the manufacturer's instructions. RNA concentration and purity were verified by spectrophotometry, and samples were stored at −80°C until analysis.

Complementary DNA (cDNA) was synthesized using the High‐Capacity cDNA Reverse Transcription Kit (Applied Biosystems). Quantitative real‐time PCR (qPCR) was carried out using TaqMan Gene Expression Assays (Thermo Fisher Scientific) for the following target genes: COL1A1 (type I collagen), COL3A1 (type III collagen), and ELN (elastin). The expression of each gene was normalized to the housekeeping gene 18S rRNA, and relative quantification was calculated using the 2^−ΔΔCt^ method.

### 2.2 Clinical Study

#### 2.2.1 Study Design

This was a prospective, single‐center, split‐hand, randomized, double‐blind clinical study evaluating CaHA diluted in PMN solution versus CaHA conventionally diluted in saline solution (SS). A single treatment session was followed by two evaluation visits at 15‐ and 90‐days post‐procedure. The study adhered to the Good Clinical Practice (GCP) guidelines and received ethics committee approval. Written informed consent was obtained from all participants before enrollment.

#### 2.2.2 Randomization and Blinding

Randomization was performed using a computer‐generated list to determine which hand would receive CaHA + PMN. The allocation information was sealed in opaque envelopes and disclosed only at the time of treatment. A research support team applied color‐coded paper bracelets to each wrist—one color for the CaHA + PMN side and another for the CaHA + SS side. A separate team, not involved in the clinical evaluations, prepared the dilutions and placed the syringes on a labeled tray matching the color codes. The injector, participants, and evaluators remained blinded to the treatment assignment.

#### 2.2.3 Eligibility Criteria

The main eligibility criteria included age ≥ 35 years and visible signs of hand aging. Subjects were excluded if they had a history of allergy or known hypersensitivity to calcium hydroxyapatite, hyaluronic acid, lidocaine, or any product component; a history of keloids, connective tissue diseases, coagulopathies, or any condition deemed unsuitable by the investigator; previous hand surgery, including sclerotherapy, or a history of hand trauma; previous hand filler treatment within the last 24 months; advanced photoaging of the hand dorsum; Raynaud's disease; or marked wrinkled skin condition or excessive skin fragility on the hands.

#### 2.2.4 Treatment Protocol

The experimental and conventional control dilutions were performed at a 1:1 ratio. The CaHA diluted in PMN solution was prepared with 1.5 mL of CaHA, 1 mL of PMN solution, and 0.5 mL of 2% lidocaine. The CaHA diluted in SS was prepared with 1.5 mL of CaHA, 1 mL of SS, and 0.5 mL of 2% lidocaine. Both mixtures resulted in a total of 3 mL of diluted CaHA.

#### 2.2.5 Injection Procedure

The dorsal hand was assessed and marked before injection. Anatomical landmarks were drawn from a proximal entry point near the wrist, extending distally to the base of the proximal phalanges, ensuring complete coverage of the dorsal area.

The dorsum of the hand comprises three layers—superficial, intermediate, and deep—separated by fascial planes. The injections were placed within the superficial layer, in the potential space between the dermis and the superficial fascia, to ensure deposition of the product in an avascular and tendon‐free plane that minimizes risk and ensures even distribution.

**FIGURE 1:**
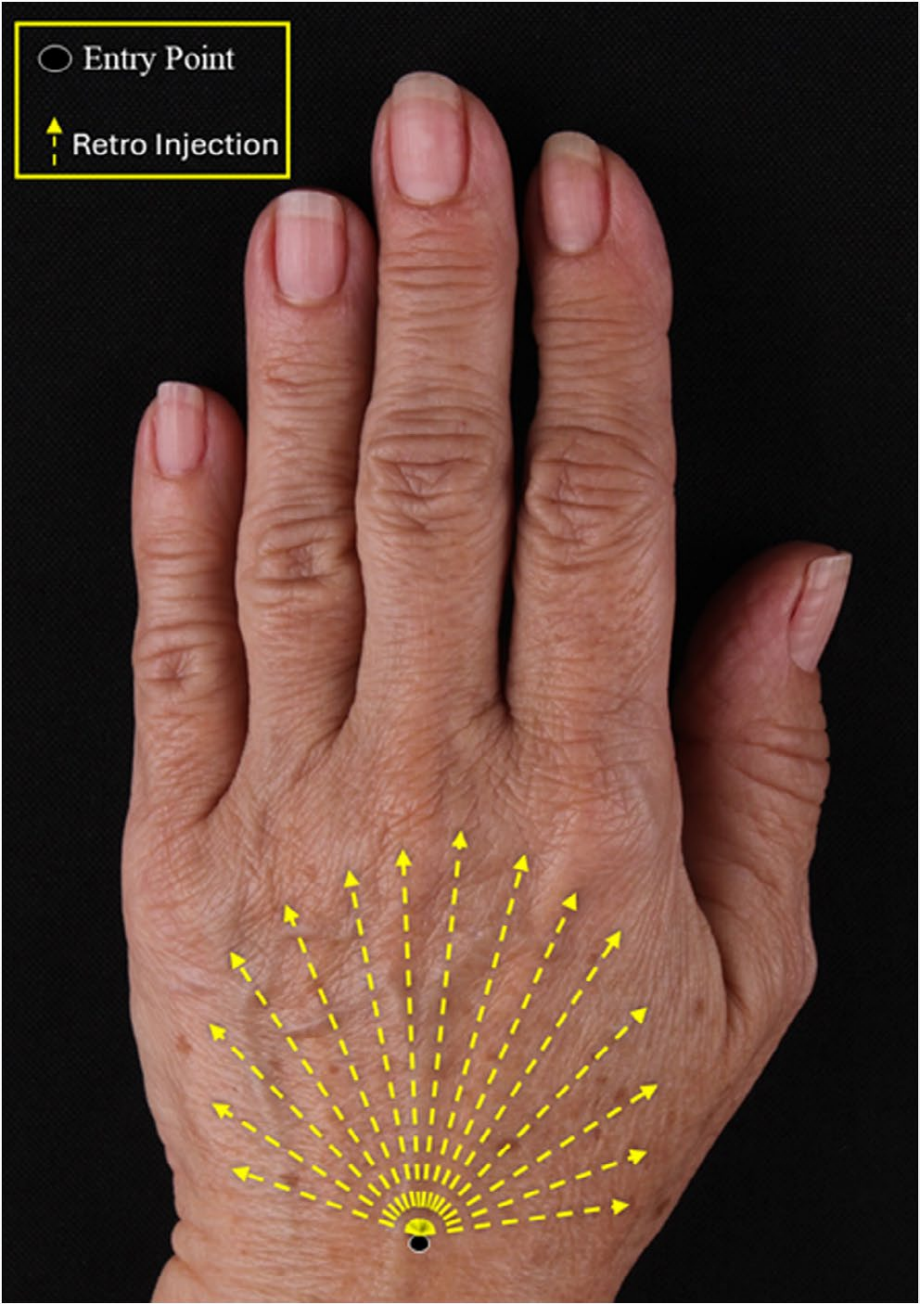
Injection technique using a fanning pattern and retro‐injections from a single‐entry point.

Each patient underwent a single treatment session. The hands were prepared with antiseptic, and local anesthesia at the entry point was administered. The procedure was performed using a 22G × 50 mm cannula. Initially, the cannula was introduced longitudinally 1 cm from the wrist and advancing in the subdermal plane (Figure 1). A total of 1.5 mL of diluted CaHA was injected per hand, following the randomization protocol, with 15 linear retroinjection passes of 0.1 mL each. Homogeneous distribution was achieved employing a fanning technique to cover the entire area to be treated.

#### 2.2.6 Outcome Assessments

All clinical and instrumental assessments were performed at baseline (D0), and at 15 (D15) and 90 (D90) post‐treatment.

##### 2.2.6.1 Efficacy and Patient Satisfaction

Aesthetic improvement was evaluated by both the patient and three blinded physicians using the Global Aesthetic Improvement Scale (GAIS), which rates overall improvement as 1 (very much improved), 2 (much improved), 3 (improved), 4 (no change), and 5 (worse) [8, 9]. Patient satisfaction for each hand was assessed independently using a five‐point Likert scale: 5 (completely satisfied), 4 (satisfied), 3 (neutral), 2 (dissatisfied), and 1 (completely dissatisfied).

The Hand Grading Scale (HGS) was evaluated by three blinded investigators at each visit, and results were compared to baseline values.

##### 2.2.6.2 Skin Hydration Analysis

Corneometry was used to assess stratum corneum hydration with the MoistureMap MM 200 device (Courage+Khazaka Electronic, Germany). The measurement output was expressed as a Gray Index, where higher values indicate greater hydration [10].

##### 2.2.6.3 Ultrasonographic Assessment

High‐frequency ultrasound imaging was performed using an Aplio i700 system (Canon Medical Systems, Japan) to evaluate dermal thickness and echogenicity of the dorsum of the hands. All scans were performed by the same experienced radiologist, blinded to treatment allocation, to ensure unbiased assessment and minimize interobserver variability.

##### 2.2.6.4 Safety

The safety profile was monitored by recording adverse events (AE) throughout the course of the study. AEs were tracked during clinical visits and through monthly follow‐up phone calls to ensure comprehensive monitoring of patient safety.

#### 2.2.7 Statistical Methods

Fibroblast proliferation results were evaluated by normalizing the untreated control at 100% and calculating the relative cell renewal percentage of each sample accordingly. For RT‐qPCR data, relative gene expression was calculated using the 2^−ΔΔCt^ method to generate fold‐change values, whereas all statistical comparisons were performed using ΔCt values.

Group comparisons for the in vitro assays were analyzed by one‐way ANOVA followed by Bonferroni post hoc test. Statistical significance was set at *p* < 0.05.

The primary clinical endpoint was the change in Hand Grading Scale (HGS) score, assessed by three blinded investigators at the 3‐month endpoint. A difference of 1 point in HGS between the hand treated with CaHA diluted in PMN and the hand treated with the conventional CaHA diluted in SS was considered clinically relevant.

To evaluate the treatment efficacy, HGS scores were analyzed using a repeated‐measures ANOVA, with treatment and time (baseline to Day 90) included as factors.

Categorical variables, including GAIS and patient satisfaction, were analyzed descriptively using frequencies and percentages. Due to the limited sample size and the high number of response categories, it was not possible to perform formal statistical comparisons.

Multivariate analysis of variance (ANOVA) was performed to explore associations between improvements observed in the different evaluated parameters. Descriptive statistics were used for GAIS, satisfaction, and safety data, which were summarized as frequency and percentage of events.

Statistical analyses were performed using R software (version 4.4.1) [11], Microsoft Excel, and GraphPad Prism 5 (GraphPad Software, USA).

## 3 Results

### 3.1 In Vitro Analysis: Fibroblast Proliferation

**FIGURE 2:**
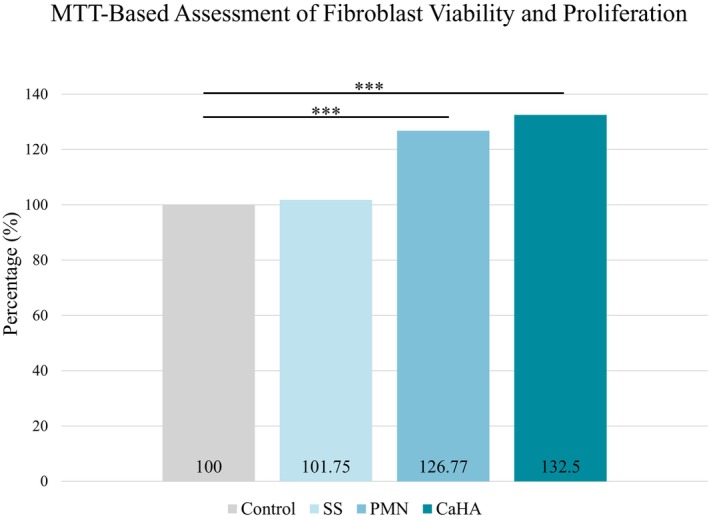
Percentage of human fibroblast viability and proliferation after exposure to isolated treatments compared to the control group (***p < 0.001).

Fibroblasts treated with CaHA or PMN alone showed increased proliferation compared with the untreated control and saline groups (Figure 2). The control group presented a baseline proliferation of 100% ± 1.89%, while saline‐treated cells showed 101.75% ± 3.09%, indicating no significant stimulatory effect. PMN alone resulted in 126.77% ± 2.56%, and CaHA alone led to 132.5% ± 2.66%, both treatments resulted in a statistically significant increase in proliferation compared to the control (*p* < 0.001).

**FIGURE 3:**
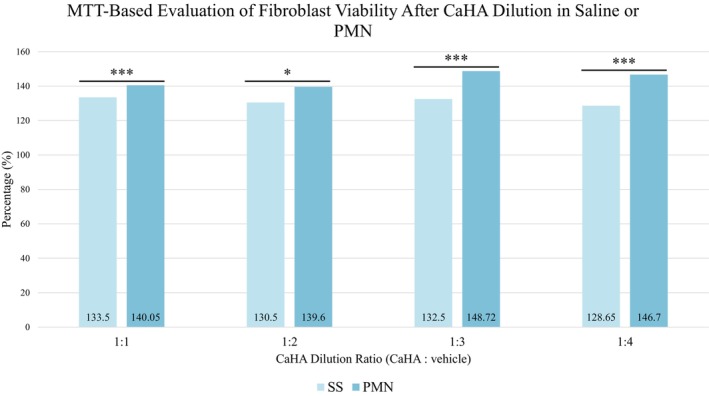
Percentage of human fibroblast viability after exposure to calcium hydroxyapatite (CaHA) diluted in saline solution (SS) or in polymicronutrient solution (PMN) across dilution ratios (1:1 to 1:4), compared to the control group (*p < 0.05; ***p < 0.001).

CaHA diluted in PMN resulted in higher cell proliferation compared to CaHA diluted in saline across all tested ratios 1:1 to 1:4 (Figure 3), reaching up to 148.72% ± 2.59%. Between‐group significance per ratio was not claimed, and results are presented as numerical differences in line with the exploratory design.

### 3.2 In Vitro Analysis: Gene Expression of Extracellular Matrix Components

**FIGURE 4:**
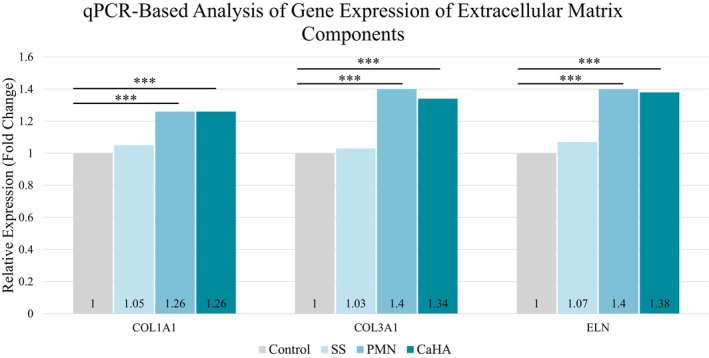
Relative mRNA expression (fold change) of COL1A1, COL3A1, and ELN in human dermal fibroblasts after 72 h exposure to saline, PMN, or CaHA. Values are normalized to control = 1 (***p < 0.001 vs control).

Fibroblasts exposed to CaHA or PMN alone showed increased expression of COL1A1, COL3A1, and ELN genes compared to the control and saline groups (Figure 4).

**FIGURE 5:**
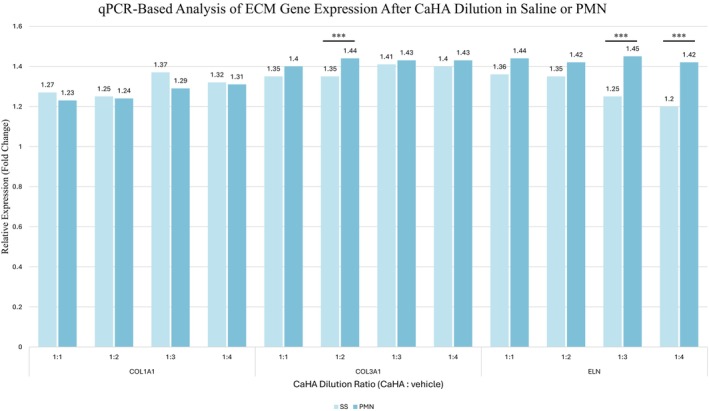
Relative mRNA expression of COL1A1, COL3A1, and ELN in human dermal fibroblasts after 72 h exposure to CaHA diluted in SS or PMN at different ratios (CaHA:vehicle = 1:1 to 1:4). Values are mean ± SD of triplicate experiments, normalized to control = 1 (***p < 0.001).

When CaHA was diluted either in saline or in the PMN solution, gene expression levels of the three proteins remained above control levels across all tested ratios (1:1 to 1:4). As shown in Figure 5, COL1A1 expression was significantly higher in the CaHA + PMN 1:2 group compared to the corresponding saline dilution (*p* < 0.001). ELN expression also showed significant up‐regulation in the 1:3 and 1:4 PMN groups (*p* < 0.001). Although other differences did not reach statistical significance, a consistent trend toward higher ECM gene expression was observed in fibroblasts treated with CaHA + PMN relative to CaHA + SS.

### 3.3 Clinical Analysis

The clinical study included 22 participants (21 women and 1 man), with a mean age of 52 ± 7.6 years. The majority (45.45%) presented HGS grade 3, corresponding to severe loss of subcutaneous tissue and moderate visibility of veins and tendons. None of the participants had a history of previous hand rejuvenation procedures.

**FIGURE 6:**
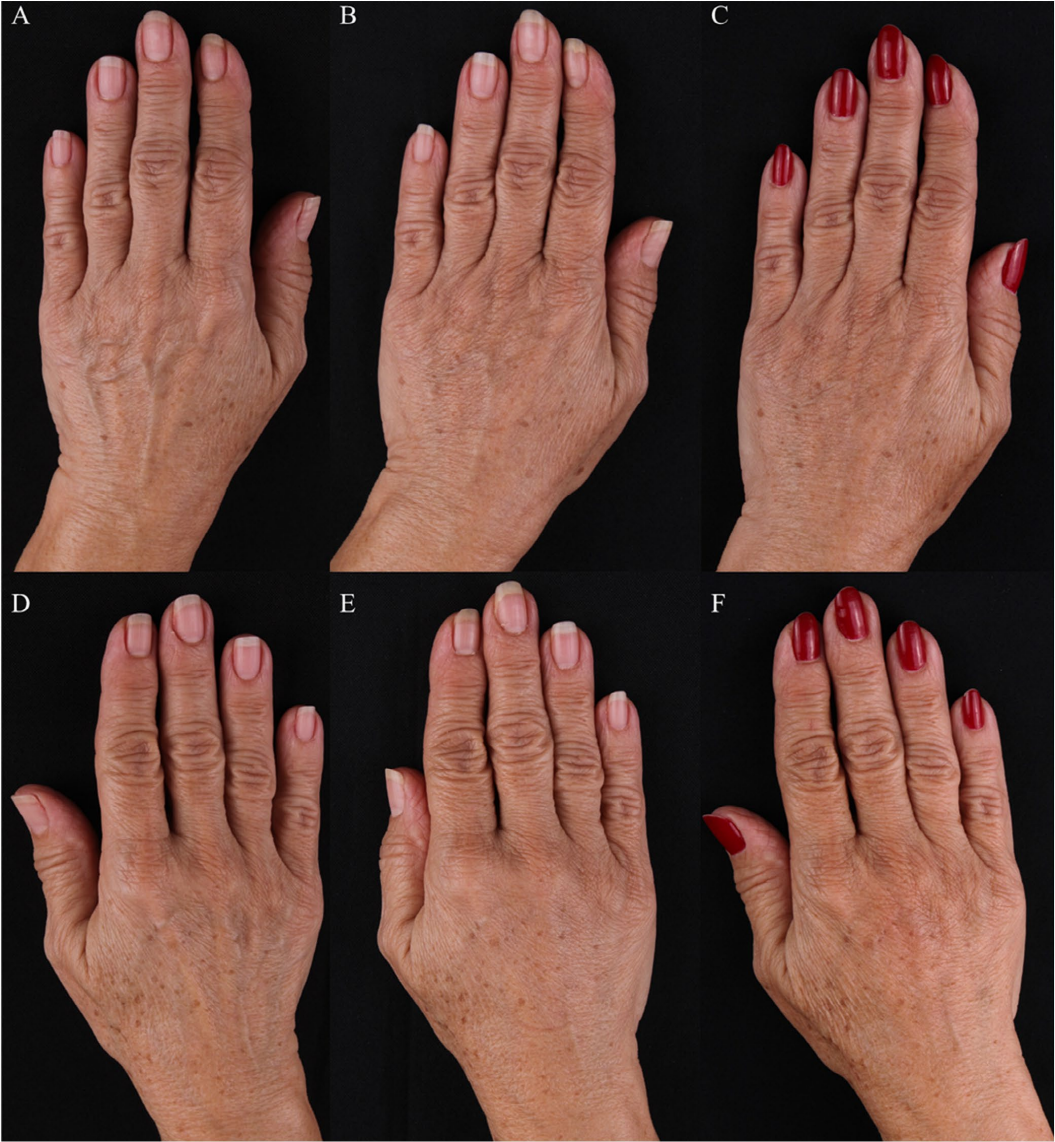
(A–F) Representative clinical photographs of dorsal hands before treatment (baseline), D15 and 90 days after injection of CaHA diluted in saline (SS) and CaHA diluted in polymicronutrient solution (PMN). (A–C) CaHA + PMN and (D–F) CaHA + SS.

**FIGURE 7:**
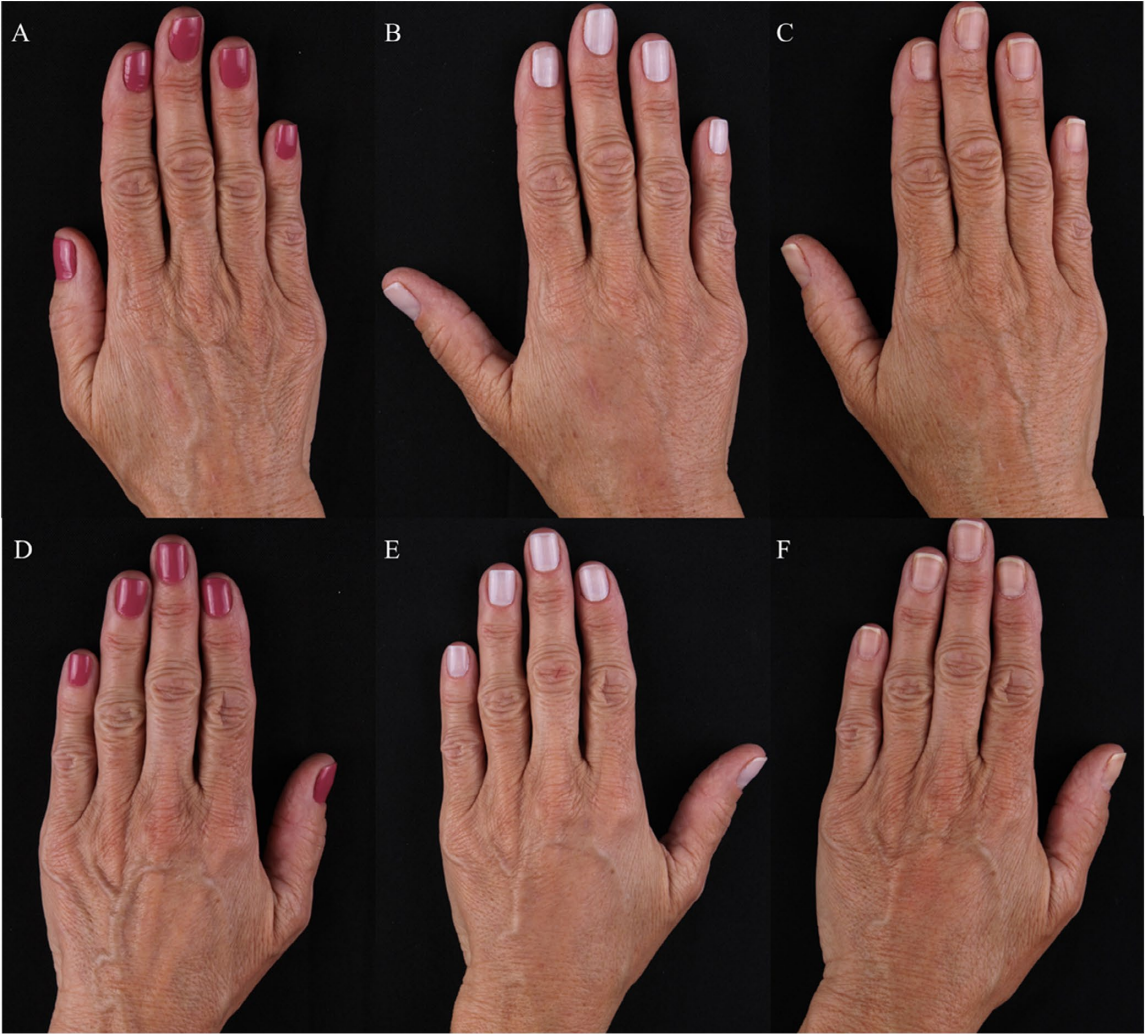
(A–F) Clinical images of right and left hands over time. The top row (A–C) shows the hand treated with calcium hydroxyapatite (CaHA) diluted in polymicronutrient solution (PMN), and the bottom row (D–F) shows the hand treated with CaHA diluted in saline solution (SS). Images were taken at baseline (A, D), Day 15 (B, E), and Day 90 (C, F).

Overall clinical improvement was observed throughout the 90‐day period, with visible enhancement in skin texture, hydration, and dermal thickness. The improvements were confirmed by standardized photographs and ultrasound imaging (Figures 6 and 7).

#### 3.3.1 Efficacy: Scores

Repeated‐measures ANOVA revealed a significant main effect of time on Hand Grading Scale (HGS) scores (*F*(2, 48) = 32.33, *p* < 0.001, ges = 0.11), indicating that HGS changed significantly across the evaluated time points (D0, D15, and D90). However, there was no significant main effect of treatment (*F*(1, 24) = 0.006, *p* = 0.937, ges = 0.0002), nor a time × treatment interaction (*F*(2, 48) = 0.15, *p* = 0.859, ges = 0.0006).

These results indicate that both CaHA + PMN and CaHA + SS treatments led to similar patterns of HGS improvement over time, with the change primarily associated with the effect of time rather than the type of dilution used. Assumptions of sphericity were met (Mauchly's *W* = 0.91, *p* = 0.33).

**FIGURE 8:**
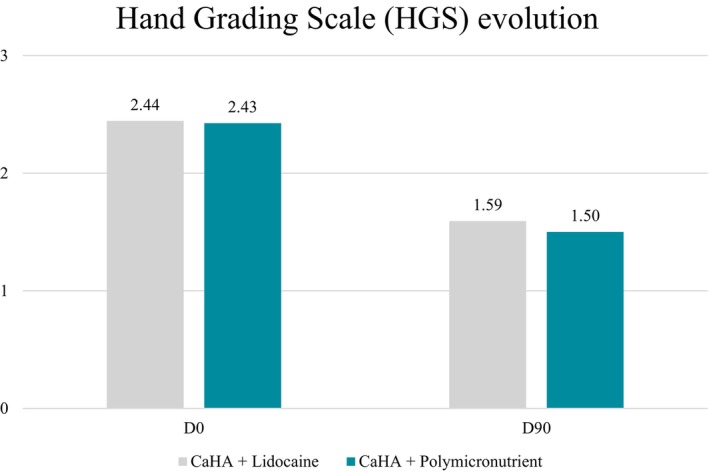
Mean change in Hand Grading Scale (HGS) from baseline to 90 days, assessed by three blinded evaluators, showing significant clinical improvement (*p* < 0.001) for both groups with no significant between‐group differences.

In the CaHA + SS group, mean HGS score decreased from 2.44 ± 0.99 at D0 to 1.59 ± 1.03 at D90, whereas in the CaHA + PMN group, HGS decreased from 2.43 ± 0.99 to 1.50 ± 0.94 (Figure 8). Both groups showed statistically significant within‐group improvement (*p* < 0.001) over time, but no between‐group difference was detected.

**FIGURE 9:**
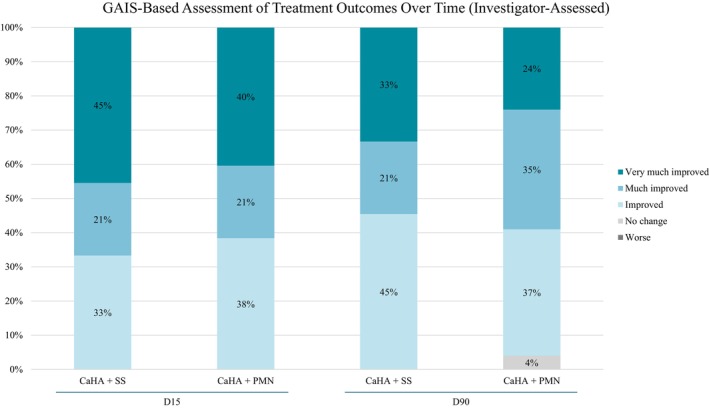
Investigator‐assessed Global Aesthetic Improvement Scale (GAIS) outcomes at D15 and D90 for both treatment groups (CaHA + SS and CaHA + PMN).

Investigator‐assessed GAIS showed that at D15, all treated hands (100%) demonstrated visible improvement in both groups. At D90, improvement was maintained in 100% of the hands treated with CaHA + SS and in 96% of those treated with CaHA + PMN (Figure 9).

**FIGURE 10:**
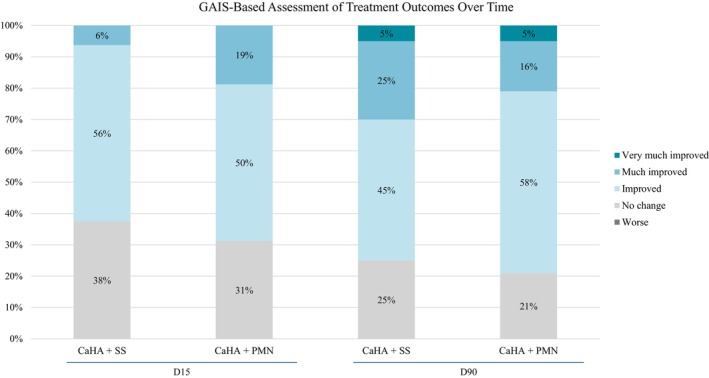
Patient‐reported Global Aesthetic Improvement Scale (GAIS) at D15 and D90 for hands treated with CaHA + SS and CaHA + PMN. Both formulations showed progressive improvement over time, with comparable overall results.

Patient‐assessed outcomes followed the same trend. At D15, improvement was reported in 62% of hands treated with CaHA + SS and 69% with CaHA + PMN. By D90, these values increased to 75% and 79%, respectively. A slightly higher proportion of patients rated the CaHA + SS hand as “very much improved” or “much improved” (30%) compared to the CaHA + PMN group (21%) (Figure 10).

Overall patient satisfaction was also assessed using the 5‐point Likert scale. Baseline dissatisfaction was comparable between groups, with 60% of hands rated as dissatisfied or completely dissatisfied. By D90, no hands were rated with dissatisfaction in either group. Satisfaction levels were descriptively higher in the CaHA + PMN group (87%) than in the CaHA + SS group (73%). However, due to the limited sample size and the distribution of responses across multiple categories, a formal statistical comparison could not be performed to determine significance.

These findings reinforce the positive perception of both treatments, confirming high acceptance and tolerability among participants, with no cases of treatment discontinuation or dissatisfaction at follow‐up.

#### 3.3.2 Skin Hydration

Skin hydration was assessed by corneometry using the MoistureMap MM 200 system (Courage + Khazaka Electronic, Germany), which measures the water content of the stratum corneum through capacitance analysis.

Both treatments resulted in a significant increase in hydration values from baseline to Day 90, confirming an overall improvement in the moisture balance of the dorsal hand skin (*p* < 0.001) (Table S3). The CaHA + PMN group showed a mean increase of 38%, while the CaHA + SS group increased by 34%, although the between‐group difference was not statistically significant (*p* = 0.294).

The Mean Gray Level (MeanGL), representing the grayscale intensity of the corneometry image, decreased in both groups over the 90 days period, indicating improved water distribution within the epidermal and superficial dermal layers. The reduction was numerically greater in the CaHA + PMN group (−10%) than in the CaHA + SS group (−8%), though without a significant intergroup difference (*p* = 0.294) (Table S3).

**FIGURE 11:**
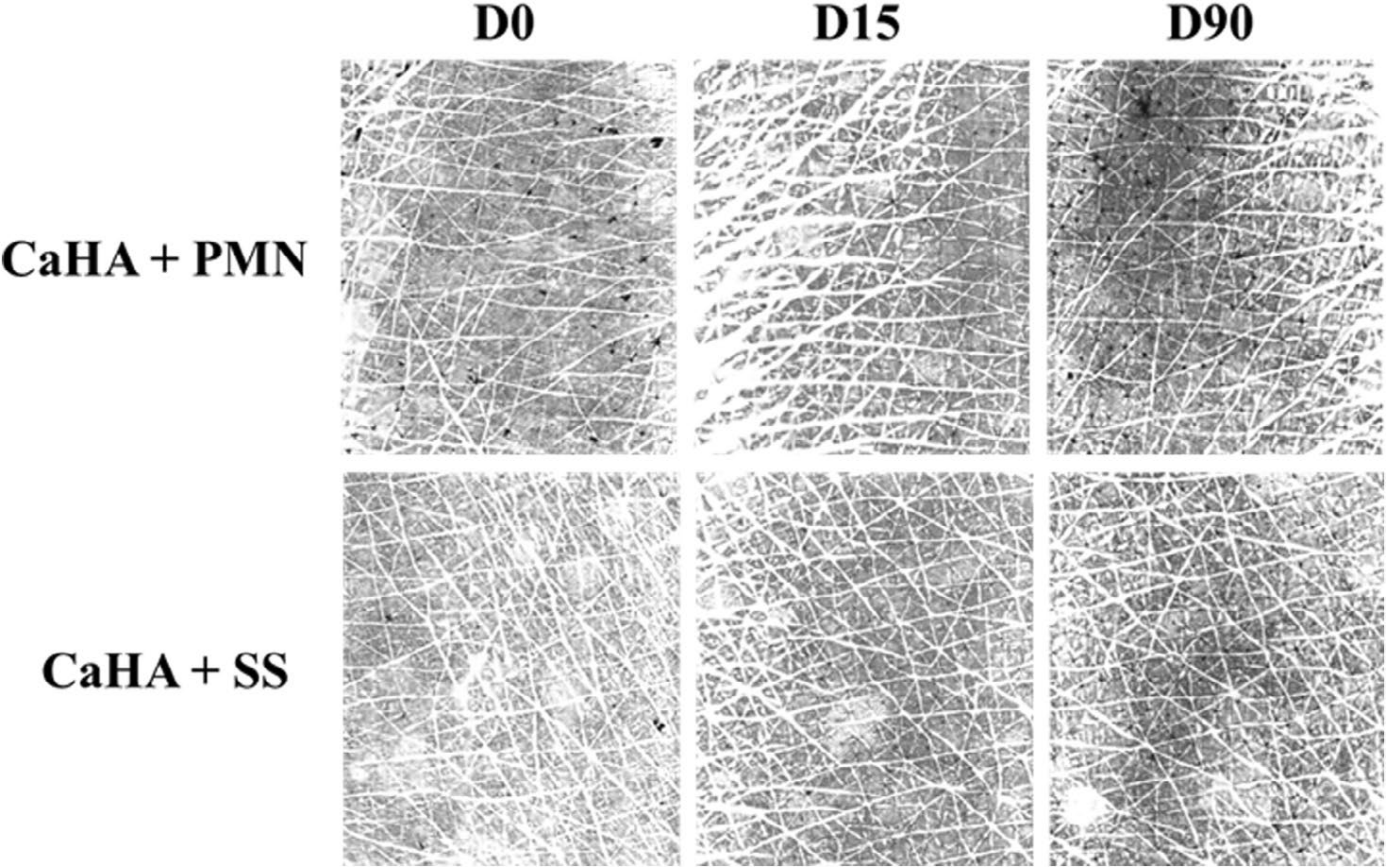
Representative corneometry (MoistureMap) images showing the hydration of the dorsal hand skin before treatment (baseline), 15 and 90 days after injection of CaHA + SS and CaHA + PMN. Both treatments demonstrated improved hydration and a more uniform water‐distribution pattern. Mean Gray Level (MeanGL) decreased and Gray Index increased in both groups.

Representative hydration map images of both treatment groups at different time points are shown in Figure 11.

#### 3.3.3 Ultrasound Imaging Measurements

Dermal and hypodermal thickness were evaluated by high‐frequency ultrasound at baseline and Day 90. Both formulations demonstrated a significant increase in dermal and hypodermal thickness over time (*p* < 0.001), confirming a measurable stimulatory effect in the treated areas.

Mean combined dermis and hypodermis layer thickness increased by 38% in the CaHA + SS group and 34% in the CaHA + PMN group. When analyzed separately, hypodermal thickness increased by 41% and 36%, respectively (Table S4). Although these within‐group increases were statistically significant, no between‐group difference was detected (*p* > 0.05).

**FIGURE 12:**
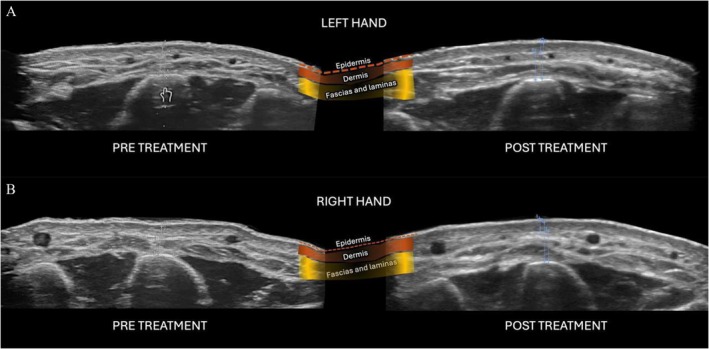
Ultrasound images pre‐ and post‐treatment (Day 90): CaHA + PMN (A) and CaHA + SS (B). Colored overlays highlight skin layers.

Qualitative ultrasound analysis demonstrated improvements in both groups (Figure 12), showing a more regular epidermal contour, along with increased dermal thickness and enhanced definition of the subcutaneous septa and laminar structures, consistent with collagen remodeling and improved tissue organization.

#### 3.3.4 Safety and Adverse Events

Both CaHA diluted in SS and CaHA diluted in PMN treatments were well tolerated, with no reports of serious adverse events throughout the study period.

The most common side effects were local reactions such as mild edema and ecchymosis, which were self‐limiting and resolved spontaneously within a few days.

A single participant developed a localized infection on both hands, which was successfully treated with oral antibiotics, with no residual effects, sequelae, or impact on treatment outcomes. No cases of nodules, granulomas, persistent inflammation, vascular compromise, or hypersensitivity reactions were observed during the follow‐up period.

Overall, both formulations demonstrated an excellent safety and tolerability profile, consistent with the established biocompatibility of CaHA‐based fillers and with previously published data on their clinical use in the hands.

## 4 Discussion

The present study integrated in vitro and clinical data to evaluate the biological and aesthetic effects of combining calcium hydroxyapatite (CaHA) with a PMN solution compared with the conventional CaHA + saline (SS) formulation for hand rejuvenation.

This dual‐arm approach provided a comprehensive understanding of the potential synergistic effect of adding metabolic cofactors to a well‐established collagen biostimulator.

Calcium hydroxyapatite (CaHA) is a well‐established and FDA‐approved option for dorsal hand rejuvenation, with its efficacy and safety consistently demonstrated across multiple clinical studies. Known for its dual mechanism of action—providing immediate volume through its gel carrier and promoting long‐term biostimulation via collagen and elastin synthesis—CaHA has become a standard treatment for addressing volume loss and skin aging in the hands. Studies have shown improvements in wrinkle severity, skin quality, patient satisfaction, and collagen density for up to 12 months after treatment, confirming both its durability and favorable safety profile [4, 12, 13].

In recent years, there has been growing interest in modifying the dilution vehicle of CaHA to enhance its regenerative effects beyond those achieved with conventional diluents such as saline or lidocaine. Various formulations—including cross‐linked hyaluronic acid fillers, free hyaluronic acid, amino acid complexes, and PMN blends—have been explored as alternative vehicles to promote tissue regeneration and optimize clinical outcomes. These combinations aim to provide either a more immediate aesthetic result, particularly when using cross‐linked HA fillers [14], or a more prominent biostimulatory effect when combined with nutrient‐rich substrates capable of supporting cellular function.

This approach, often referred to as *skin priming*, was proposed by Theodorakopoulou et al. [6], who suggested that enriching the dermal microenvironment with essential cofactors may improve fibroblast activity and support ECM remodeling. Similarly, Prikhnenko (2015) reviewed preclinical data showing that polycomponent mesotherapy formulations can enhance fibroblast proliferation and both intracellular and extracellular collagen synthesis—particularly in aged cells—thereby supporting the rationale for metabolic support in regenerative strategies [7]. In aging or metabolically compromised skin, this strategy may amplify the biostimulatory effects of CaHA by restoring the regenerative potential of local cells.

Although the theoretical advantages of combining CaHA with bioactive or hybrid diluents are compelling, current clinical evidence remains limited. Most available studies rely on anecdotal reports, small case series, or non‐comparative designs, which hinder the ability to establish whether these combinations truly offer superior outcomes compared to conventional dilution with saline or lidocaine. Additionally, the underlying biological mechanisms—particularly their impact on fibroblast activity and ECM remodeling—remain poorly understood. To address these gaps, we conducted a split‐hand, randomized, double‐blind clinical trial designed to explore clinical and biological differences between CaHA diluted in a PMN solution versus saline. In parallel, we performed in vitro experiments simulating clinical formulations to investigate their effects on fibroblast proliferation and gene expression of ECM components. This dual approach allowed for the simultaneous investigation of biological plausibility and clinical performance under controlled conditions.

In vitro results demonstrated that both CaHA and PMN, when used individually, significantly increased fibroblast proliferation compared to untreated control. When combined, CaHA diluted in PMN induced a greater proliferative effect than dilution in saline, with statistically significant differences observed at all tested ratios. Gene expression analysis revealed significant upregulation of COL3A1 and ELN in specific PMN dilution groups compared to saline. Although not all comparisons reached statistical significance, a consistent trend toward increased expression of ECM components was observed in the PMN groups. However, as with any in vitro model, limitations exist: the controlled environment lacks the complex interactions of living tissue, including immune system influence, and vascularization. Additionally, the relatively short treatment duration may not fully reflect long‐term effects, and fibroblasts from healthy donors may respond differently than those from aged or metabolically compromised skin.

Clinically, both formulations—CaHA diluted in saline (SS) and CaHA diluted in PMN—resulted in notable improvements in hand appearance, as evidenced by reductions in Hand Grading Scale (HGS) scores, increased hydration and greater dermal and hypodermal thickness on ultrasound imaging. These findings reinforce the well‐documented capacity of CaHA to induce structural remodeling and restore volume in aging hands. Ultrasound imaging further supported these effects by demonstrating increased dermal and hypodermal thickness in both groups, with no statistically significant difference between hands treated with CaHA + SS and those treated with CaHA + PMN.

While no safety concerns were observed, the off‐label (intradermal or subdermal injection) use of the study's PMN in clinical practice should be approached with caution by professionals aware of its current regulatory limitations.

Some limitations of this study should be acknowledged. First, although the PMN solution showed promising biological activity in vitro, clinical outcomes such as HGS, hydration, and ultrasound did not demonstrate statistically significant differences between groups. This apparent disconnect between cellular‐level findings and comparable clinical outcomes may be explained by multiple factors. One possible explanation is the relatively low injection volume of PMN, which may have limited its clinical impact. Another is the strong and well‐established biostimulatory effect of CaHA, which could have overshadowed subtle additive benefits of the nutrient‐rich vehicle. Moreover, the clinical endpoints applied, although validated, may not be sensitive enough to detect subtle regenerative changes, and the 90‐day follow‐up might have been insufficient to capture long‐term effects. In addition, the limited sample size, absence of power calculation, and relatively short follow‐up may have reduced the ability to detect small but potentially meaningful clinical differences. To better understand the clinical potential of PMN as a priming agent, future research should focus on larger sample sizes and longer follow‐up periods (e.g., 6–12 months) to determine if regenerative differences emerge over time. Furthermore, investigating varying concentrations of PMN and alternative dilution ratios (such as 1:2 or 1:3) may help identify an optimal dose–response relationship that was not fully captured in the current 1:1 clinical protocol. Exploring the effect of multiple PMN sessions prior to CaHA injection could also provide insights into the ‘skin priming’ hypothesis. In parallel, mechanistic studies in aged skin tissue and more sensitive outcome measures may help detect subtle regenerative changes.

## 5 Conclusion

This study demonstrated that both CaHA diluted in saline (CaHA + SS) and CaHA diluted in a PMN solution (CaHA + PMN) are effective and safe for hand rejuvenation.

Clinical and instrumental assessments confirmed significant improvements in hand contour, hydration, and dermal thickness in both treatment groups.

While the CaHA + PMN formulation induced greater fibroblast proliferation and ECM gene upregulation in vitro, these biological differences did not translate into clinically significant superiority within the 3‐month observation period.

Both formulations achieved comparable aesthetic and patient satisfaction outcomes, with excellent tolerability and no serious adverse events. However, these findings should be interpreted considering the study's limited sample size, absence of power calculation, and short‐term follow‐up. Taken together, these findings support the safety and efficacy of CaHA for dorsal hand rejuvenation and highlight the potential of polymicronutrient enrichment as a promising complementary strategy for enhancing fibroblast metabolic activity.

Further studies with larger sample sizes and longer follow‐up periods are warranted to determine whether the biological advantages observed in vitro translate into sustained clinical benefit over time.

## Author Contributions

G.E.L.F., C.H., R.M.M.V., B.D.O., R.I.S.M., and R.F.B. contributed to the study design. G.E.L.F., B.D.O., R.M.M.V., R.I.S.M., and A.C.H.R.M. were responsible for methodology development. Clinical case management and data collection were performed by G.E.L.F., B.D.O., R.I.S.M., R.M.M.V., A.C.H.R.M., and L.Z. Data analysis was conducted by G.E.L.F., C.H., R.M.M.V., B.D.O., R.I.S.M., A.C.H.R.M., and L.Z. The manuscript was written by G.E.L.F., R.M.M.V., and B.D.O. All authors contributed to the critical review of the manuscript and approved the final version for submission.

## Ethics Statement

This study was reviewed and approved by the appropriate institutional review board (CAAE: 76910323.4.0000.5450; approval number: 6.698.463). Written informed consent for participation and publication was obtained from all participants, with the understanding that the information may be made publicly available.

## Conflicts of Interest

Beatriz Domenici de Oliveira is an employee of Ilikia Brasil, and Renata Viana serves as a scientific consultant for Ilikia Brasil. Ilikia Brasil had no role in the study design; data collection, analysis, or interpretation; or the decision to submit the manuscript for publication. The remaining authors declare no conflicts of interest.

## Supporting information


**Table S1:** Polymicronutrients contained in a 5 mL vial of Pluryal Mesoline Refresh.
**Table S2:** Summary of treatment groups and calcium hydroxyapatite (CaHA) dilution ratios used in cell culture experiments.
**Table S4:** Ultrasound image measurements from baseline to Day 90 for calcium hydroxyapatite (CaHA) diluted in saline solution (SS) and in polymicronutrient solution (PMN).

**Table S3:** Skin hydration outcomes from baseline to Day 90 for calcium hydroxyapatite (CaHA) diluted in saline solution (SS) and in polymicronutrient solution (PMN).

## Data Availability

The data that support the findings of this study are available from the corresponding author upon reasonable request.
