# Supplementary material for: Calcium Hydroxyapatite and Polymicronutrient Solution on Hand Rejuvenation: A Split‐Hand, Randomized, Double‐Blind Clinical and In Vitro Study
Source: J Cosmet Dermatol. 2026 Feb 11;25(2):e70716. doi: 10.1111/jocd.70716 (PMC12892161; doi:10.1111/jocd.70716)
Supplement: Supplementary file 1 — Table S1: Polymicronutrients contained in a 5 mL vial of Pluryal Mesoline Refresh. Table S2: Summary of treatment groups and calcium hydroxyapatite (CaHA) dilution ratios used in cell culture experiments. Table S4: Ultrasound image measurements from baseline to Day 90 for calcium hydroxyapatite (CaHA) diluted in saline solution (SS) and in polymicronutrient solution (PMN). [file JOCD-25-e70716-s002.docx]

**Supplementary**

**Supplem. Material, Table 1**. Polymicronutrients contained in 5ml vial of Pluryal® Mesoline Refresh.

| **Component** |
| --- |
| Hyaluronic acid (50mg) |
| Sodium Chondroitin Sulfate |
| Polynucleotides from salmon DNA |
| Plant-based Stem Cell Enhancer |
| Panthenol (Vitamin B5) |
| Pyridoxine |
| Niacin |
| Retinoyl Palmitate (Vitamin A) |

**Supplem. Material, Table 2**. Summary of treatment groups and calcium hydroxyapatite (CaHA) dilution ratios used in cell culture experiments.

| **Treatment** | **Dilution ratio (CaHA : vehicle)** |
| --- | --- |
| Negative control (untreated) | — |
| Saline Solution only | — |
| CaHA only | — |
| PMN only | — |
| CaHA + SS | 1:1 |
| CaHA + SS | 1:2 |
| CaHA +SS | 1:3 |
| CaHA + SS | 1:4 |
| CaHA + PMN | 1:1 |
| CaHA + PMN | 1:2 |
| CaHA + PMN | 1:3 |
| CaHA + PMN | 1:4 |

**Supplem. Material, Table 3:** Skin hydration from baseline to 90 days of tested treatments, calcium hydroxyapatite (CaHA) diluted in saline solution (SS) and in polymicronutrient solution (PMN).

| **Parameter** | **Treatment** | **D0 Mean** | **D0**  **SD** | **D90 Mean** | **D90**  **SD** | **Diff between means** | **% of variation** | **p-value treatment** | **p-value time** |
| --- | --- | --- | --- | --- | --- | --- | --- | --- | --- |
| GreyIdx (Grey Index) | CaHA + SS | 59.0 | 18.4 | 79.2 | 15.9 | 20.2 | 34 | 0.294 | <0.001 |
|  | CaHA + PMN | 59.6 | 17.8 | 82.5 | 19.9 | 22.9 | 38 |  |  |
| MeanGL (Mean Grey Level) | CaHA + SS | 213 | 20 | 195 | 16 | -18 | -8 | 0.294 | <0.001 |
|  | CaHA + PMN | 212 | 20 | 190 | 19 | -22 | -10 |  |  |

**Supplem. Material, Table 4:** Ultrasound image measurements of tested treatments, calcium hydroxyapatite (CaHA) diluted in saline solution (SS) and in polymicronutrient solution (PMN).

| **Parameter** | **Treatment** | **D0**  **Mean** | **D0**  **SD** | **D90**  **Mean** | **D90**  **SD** | **Diff between means** | **% of variation** | **p-value treatment** | **p-value time** |
| --- | --- | --- | --- | --- | --- | --- | --- | --- | --- |
| Epidermis/dermis (mm) | CaHA + SS | 1.06 | 0.21 | 1.24 | 0.29 | 0.18 | 17 | 0.202 | 0.001 |
|  | CaHA + PMN | 0.94 | 0.19 | 1.20 | 0.22 | 0.26 | 27 |  |  |
| Dermis and hypodermis (mm) | CaHA + SS | 2.86 | 0.75 | 3.94 | 0.78 | 1.08 | 38 | 0.581 | <0.001 |
|  | CaHA + PMN | 3.01 | 0.80 | 4.03 | 0.77 | 1.02 | 34 |  |  |
| Hypodermis (mm) | CaHA + SS | 2.16 | 0.76 | 3.04 | 0.86 | 0.88 | 41 | 0.510 | <0.001 |
|  | CaHA + PMN | 2.33 | 0.83 | 3.16 | 0.80 | 0.83 | 36 |  |  |
