# Supplementary material for: Calcium Hydroxyapatite and Polymicronutrient Solution on Hand Rejuvenation: A Split‐Hand, Randomized, Double‐Blind Clinical and In Vitro Study
Source: J Cosmet Dermatol. 2026 Feb 11;25(2):e70716. doi: 10.1111/jocd.70716 (PMC12892161; doi:10.1111/jocd.70716)
Supplement: Supplementary file 2 — Table S3: Skin hydration outcomes from baseline to Day 90 for calcium hydroxyapatite (CaHA) diluted in saline solution (SS) and in polymicronutrient solution (PMN). [file JOCD-25-e70716-s001.pptx]

## Slide 1
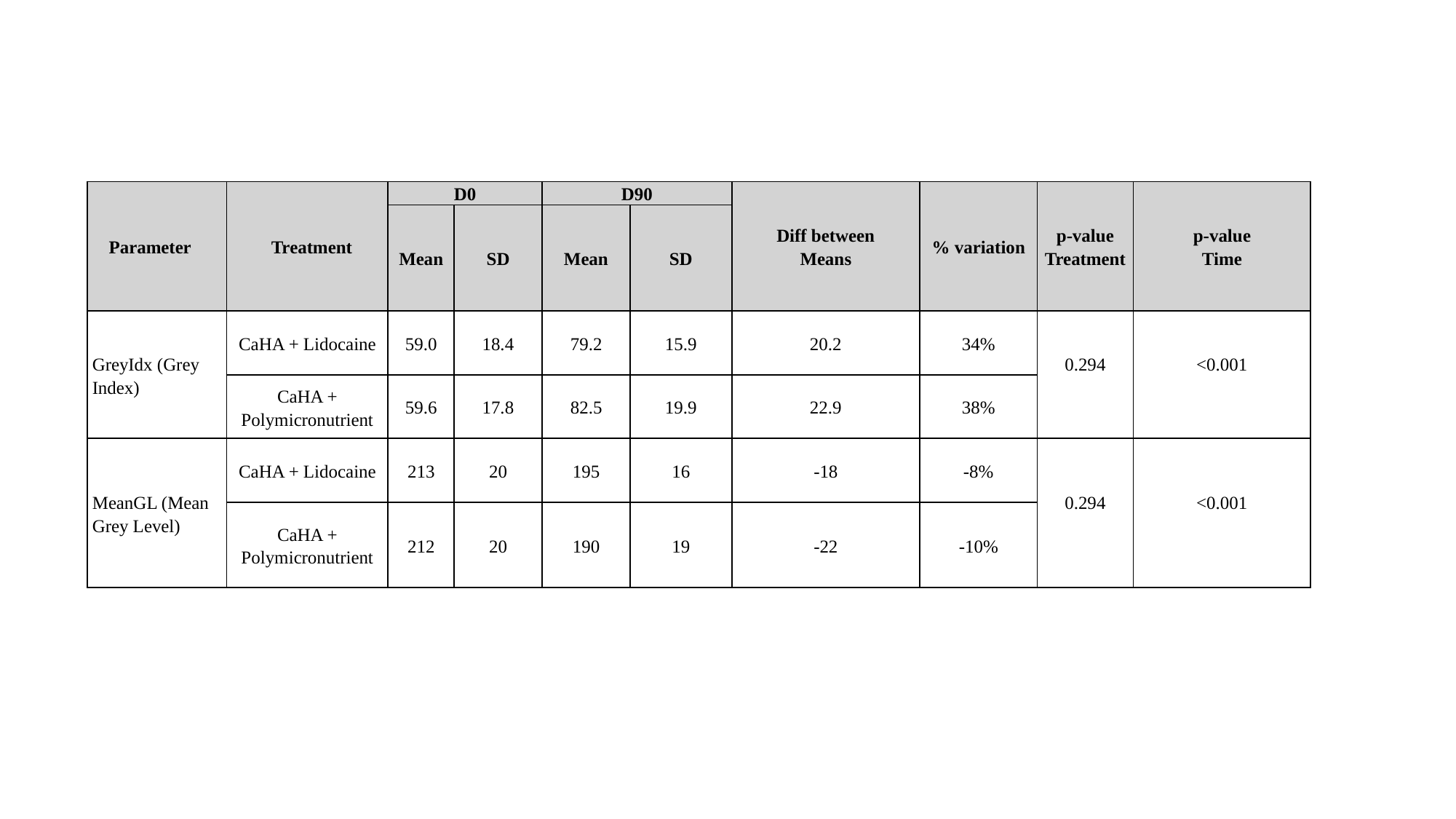

| Parameter | Treatment | D0 | | D90 | | Diff between Means | % variation | p-value Treatment | p-value Time |
| --- | --- | --- | --- | --- | --- | --- | --- | --- | --- |
| | | Mean | SD | Mean | SD | | | | |
| GreyIdx (Grey Index) | CaHA + Lidocaine | 59.0 | 18.4 | 79.2 | 15.9 | 20.2 | 34% | 0.294 | <0.001 |
| | CaHA + Polymicronutrient | 59.6 | 17.8 | 82.5 | 19.9 | 22.9 | 38% | | |
| MeanGL (Mean Grey Level) | CaHA + Lidocaine | 213 | 20 | 195 | 16 | -18 | -8% | 0.294 | <0.001 |
| | CaHA + Polymicronutrient | 212 | 20 | 190 | 19 | -22 | -10% | | |
